# Association between hospital ownership and patient selection, management, and outcomes after carotid endarterectomy or carotid artery stenting – Secondary data analysis of the Bavarian statutory quality assurance database –

**DOI:** 10.1186/s12893-024-02448-6

**Published:** 2024-05-17

**Authors:** Andreas Kuehnl, Michael Kallmayer, Bianca Bohmann, Vanessa Lohe, Rebecca Moser, Shamsun Naher, Felix Kirchhoff, Hans-Henning Eckstein, Christoph Knappich

**Affiliations:** 1 Department for Vascular and Endovascular Surgery, Klinikum rechts der Isar, Technical University of Munich, Ismaninger Str. 22, 81675 Munich, Germany; 2 Landesarbeitsgemeinschaft zur datengestützten, einrichtungsübergreifenden Qualitätssicherung in Bayern, Munich, Germany

**Keywords:** Hospital ownership, Quality assurance, Carotid stenosis, Carotid endarterectomy, Carotid stenting

## Abstract

**Background:**

This study analyses the association between hospital ownership and patient selection, treatment, and outcome of carotid endarterectomy (CEA) or carotid artery stenting (CAS).

**Methods:**

The analysis is based on the Bavarian subset of the nationwide German statutory quality assurance database. All patients receiving CEA or CAS for carotid artery stenosis between 2014 and 2018 were included. Hospitals were subdivided into four groups: university hospitals, public hospitals, hospitals owned by charitable organizations, and private hospitals. The primary outcome was any stroke or death until discharge from hospital. Research was funded by Germany’s Federal Joint Committee Innovation Fund (01VSF19016 ISAR-IQ).

**Results:**

In total, 22,446 patients were included. The majority of patients were treated in public hospitals (62%), followed by private hospitals (17%), university hospitals (16%), and hospitals under charitable ownership (6%). Two thirds of patients were male (68%), and the median age was 72 years. CAS was most often applied in university hospitals (25%) and most rarely used in private hospitals (9%). Compared to university hospitals, patients in private hospitals were more likely asymptomatic (65% vs. 49%). In asymptomatic patients, the risk of stroke or death was 1.3% in university hospitals, 1.5% in public hospitals, 1.0% in hospitals of charitable owners, and 1.2% in private hospitals. In symptomatic patients, these figures were 3.0%, 2.5%, 3.4%, and 1.2% respectively. Univariate analysis revealed no statistically significant differences between hospital groups. In the multivariable analysis, compared to university hospitals, the odds ratio of stroke or death in asymptomatic patients treated by CEA was significantly lower in charitable hospitals (OR 0.19 [95%-CI 0.07–0.56, *p* = 0.002]) and private hospitals (OR 0.47 [95%-CI 0.23–0.98, *p* = 0.043]). In symptomatic patients (elective treatment, CEA), patients treated in private or public hospitals showed a significantly lower odds ratio compared to university hospitals (0.36 [95%-CI 0.17–0.72, *p* = 0.004] and 0.65 [95%-CI 0.42-1.00, *p* = 0.048], respectively).

**Conclusions:**

Hospital ownership was related to patient selection and treatment, but not generally to outcomes. The lower risk of stroke or death in the subgroup of electively treated patients in private hospitals might be due to the right timing, the choice of treatment modality or actually to better structural and process quality.

**Supplementary Information:**

The online version contains supplementary material available at 10.1186/s12893-024-02448-6.

## Introduction

In contrast to tax-financed national health systems (Beveridge-model) such as in England, Spain, Denmark or the Scandinavian countries, the German health care system is based on social-insurance contributions (Bismarck-model) [1]. Embedded in extensive social legislation frameworks, German hospitals are deliberately exposed to the forces of the free market economy. As a result, the German hospital landscape is characterized by a wide variety of hospital structures and ownership. In addition to universities, also municipalities, charities, churches, and profit-oriented private companies are hospital owners. For example, in 2020, 29% of German hospitals were publicly owned, 33% were non-profit, and 38% privately owned [2]. A detailed overview of the development of the German hospital structure and hospital remuneration over the last 100 years can be found in the publication by Jeurissen et al. [3]. Since 2003, the German hospital reimbursement is based on a case flat-rate system (Diagnosis related groups, DRG) and therefore the question arises whether economic constraints of owners and return-on-investment expectations of investors have an influence on patient selection (e.g. cherry-picking), care, and outcomes [4–7].

The effect of hospital ownership on structures, processes, and outcomes were already analysed in a couple of studies with respect to, for example, heart failure hospitalization [8], outcomes after treatment for acute myocardial infarction [9], postoperative complications [10], left ventricular assist devices [11], hospital mortality [12], and healthcare-associated infection rates [13]. With respect to carotid stenosis – which is a common cause of ischemic stroke – a secondary data analysis of the Nationwide Inpatient Sample by Chandler et al. revealed that for-profit hospital ownership was associated with a higher rate of carotid artery stenting (CAS) compared to non-profit hospitals [14]. Unfortunately, no data on outcomes were reported. In addition to other causes, a provider-induced effect does not seem unreasonable even for carotid revascularisation.

In Germany, carotid endarterectomy (CEA) and CAS are subject to nationwide statutory quality assurance [15]. In addition to the primary purpose of quality assurance, these data can also be used for scientific analyses. Among other things, the effects of age, sex, annual hospital volume, and surgical techniques on outcomes have already been studied by our research group [16–26].

To the best of our knowledge, there is no study addressing the relationship between hospital ownership and the outcomes of carotid revascularisation. Thus, in this study, the association between hospital ownership and patient selection, treatment, and outcomes of CEA and CAS were analysed.

## Methods

The present analysis is a pre-planned sub study of the ISAR-IQ project (Integration and Spatial Analysis of Regional, Site-specific, and patient-level factors for Improving Quality of treatment for carotid artery stenosis).

### Data source

This study is based on the Bavarian subset of the nationwide German statutory quality assurance measures according to § 136 SGB V of the Federal Joint Committee operated by the Institute for Quality Assurance and Transparency in Healthcare (Institut für Qualitätssicherung und Transparenz im Gesundheitswesen, IQTIG). Data on carotid revascularisation procedures (CEA and CAS) in Bavarian Hospitals have been statutorily collected by the Bayerische Arbeitsgemeinschaft Qualitätssicherung (BAQ) and passed to the IQTIG. Because of legal obligations, the data collection covers all CEA operations and CAS procedures, except of military hospitals and out-patient clinics. Thus, the documentation is complete for all CEA and CAS, as all hospitals must report procedures. The study was approved by the Ethics Committee of the Medical Faculty, Technical University of Munich (Reference Number 107/20S). The analysis was conducted according to Good Practice of Secondary Data Analysis guidelines [27]. As this is an observational study using routinely collected health data, RECORD reporting guidelines were applied as appropriate [28]. All data are saved on BAQ servers, according to the respective data protection regulations. Data access was only permitted using controlled on-site data processing. The ISAR-IQ study protocol was submitted to the BAQ, the IQTIG and the G-BA during the application procedure but was not published separately. Further details on methods were already published [16–19, 21–26, 29, 30].

### Inclusion and exclusion criteria

**Fig. 1:**
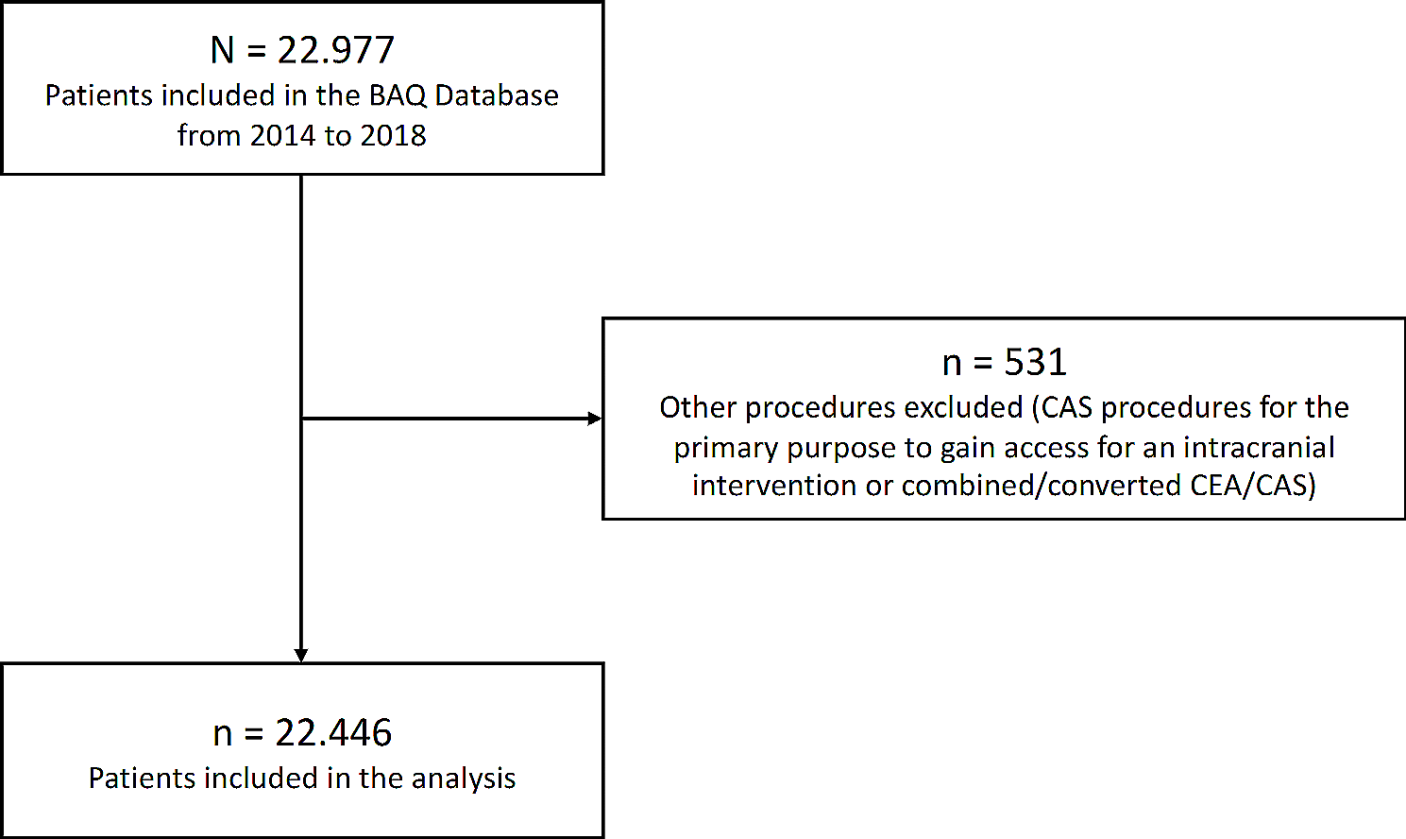
Patient flowchart. BAQ = Bayerische Arbeitsgemeinschaft für Qualitätssicherung (Bavarian Working Group for Quality Assurance), CEA = Carotid endarterectomy, CAS = Carotid artery stenting

All patients receiving either CEA or CAS for carotid stenosis (asymptomatic, symptomatic, emergency, simultaneous operation, other indications) between 2014 and 2018 in Bavarian hospitals were included (*N* = 22,977). 531 Patients with CAS procedures for the primary purpose to gain access for an intracranial intervention or combined/converted CEA/CAS procedures were excluded. In addition, patients with unknown or diverse sex were excluded. The latter was necessary to avoid extensive output blocking due to data protection issues. Please see Fig. 1 for flow chart. Patients were subdivided according to neurological symptoms: asymptomatic patients (no carotid-related cerebral or ocular symptoms within the last 6 months, indication group A), symptomatic patients receiving elective treatment (indication group B), and symptomatic patients receiving emergency treatment, simultaneous operations or other procedures (indication group C).

### Study variables

The main variable of this study was type of hospital ownership. Hospitals were subdivided into four groups: university hospitals (Group 1), public hospitals (Group 2), hospitals owned by charitable organizations (Group 3), and private for-profit hospitals (Group 4). The categorisation was mutual exclusive, so there was no overlap. Information about ownership was derived from the German hospital directory and annual mandatory quality reports. Information was cross-checked with homepages and direct mailing to the hospitals.

Patient level (first level) data were available directly from the statutory quality assurance database. These datasets were linked to hospital level data (second level), and regional data (third level) using a unique statutory hospital identifier (IK, Institutionskennzeichen) and standardized identifiers of European regions (NUTS level 3, Nomenclature des Unités territoriales statistiques). Locations and identifiers of hospitals (IK in combination with site number) were individually verified using hospital directory, mandatory quality reports, homepages, and request for final data check by the hospital itself. For data protection reasons, the unique hospital identifier is pseudonymised when the data are transmitted to the IQTIG, so that linking the level 1 data with those of levels 2 and 3 was unfortunately only possible for Bavaria. Second level data (hospital level) were gathered from mandatory quality reports, hospital directory, the German Vascular Society (certified vascular centers), and the German Stroke Society (certified stroke centers). Third level data (regional data) were derived from the German statistical office, the INKAR database (Indikatoren und Karten zur Raum- und Stadtentwicklung), and the National Association of Statutory Health Insurance Physicians (Kassenärztliche Bundesvereinigung).

### Study outcomes

The primary outcome of this study was any stroke or death until discharge from hospital. Secondary outcomes were in-hospital rates of major stroke or death, any stroke, all-cause death, myocardial infarction and major adverse cardiovascular events (MACE, combined rate of any stroke, death or myocardial infarction), and total length of hospital stay.

### Statistical analyses

Categorical variables were presented as absolute numbers and percentages. Continuous variables were uniformly presented as median with first (Q1) and third (Q3) quartiles. To analyse differences between hospital groups, three different regression models were fitted. First, a model containing only the hospital group as variable (‘univariate’ model). Random effects estimates were calculated using Mantel-Haenszel statistics with sample size weighting. Second, to account for confounding, a generalised linear model was fitted, containing the variables age, sex, ASA, ipsi- and contralateral degree of stenosis, pre- and post-procedural assessment by neurologist, and annual caseload of treating centre (extended model). For indication group B, neurological symptoms at presentation and time interval between index event and treatment were additionally entered into the model. Model specification and variable selection were done a priori according to a prespecified analysis plan based on literature research and expert knowledge. Since the number of outcome events was small, the formation of an extended model was considered overfitted. Thus, based on previous analyses of our research group [17–26] and results from the abovementioned extended model analysis, only the factors preprocedural and postprocedural examination by a neurologist were integrated into a third model (basic model), as these were the strongest confounders. All variables were entered as fixed-effect factors. Interpretation of Akaike information criterion (AIC) was in accordance to Burnham and Anderson [31].

R version 4.2.2 (R Foundation for Statistical Computing, Vienna, Austria) was used for data processing and statistical analysis, with extension packages *tidyverse*, *epitools* and *ggplot2* to calculate cross-classified tables, chi-square tests, and multivariable regression analyses. Variable codes were extracted from the codebooks provided by the IQTIG Institute and harmonized over the time period from 2005 to 2018. Graphic processing of the data was performed using Microsoft Excel. For all tests, a two-tailed level of significance of α = 5% was used.

## Results

### Characteristics of patients

In total, 22,446 patients were included (see Table 1). The majority of patients were treated in public hospitals (62%), followed by private hospitals (17%), university hospitals (16%), and hospitals under charitable ownership (6%). Two thirds of patients were male (68%), with a median age of 72 years. The therapy was performed in approximately equal parts on the right (50.5%) and left side (49.5%). CEA was performed in 82% of patients. CAS was most often applied in university hospitals (25%) and most rarely used in private hospitals (9%). Most patients were ASA stage III (61%) followed by ASA stage I/II (33%). Patients in private hospitals were classified sicker (ASA I/II 23%, ASA III 72%) compared to university hospitals (ASA I/II 36%, ASA III 57%). More than half of patients were asymptomatic (55%). One third of patients were symptomatic (elective treatment, 34%) or suffered from other conditions (e.g. emergency treatment for stroke-in-evolution or crescendo-TIA (11%)). Compared to university hospitals, patients in private hospitals were more likely asymptomatic (65% vs. 49%). For further details, see Table 1.

**Table 1:** Characteristics of patients

	Hospital ownership								all patients	
	University		Public		Charitable		Private			
**Total number of patients** (row-%)	3565	(16)	13,865	(62)	1262	(6)	3754	(17)	22,446	(100)
**Age (years, median, Q1–3)**	71	(63–77)	73	(65–78)	72	(65–77)	73	(66–78)	72	(65–78)
**Male sex**	2461	(69)	9357	(67)	869	(69)	2544	(68)	15,231	(68)
**Right carotid artery treated**	1796	(50)	6988	(50)	632	(50)	1913	(51)	11,329	(51)
**Treated by CAS**	909	(25)	2551	(18)	145	(11)	348	(9)	3953	(18)
**ASA stage** ^$^										
Stage I + II	1301	(36)	4890	(35)	418	(33)	857	(23)	7466	(33)
Stage III	2033	(57)	8278	(60)	785	(62)	2708	(72)	13,804	(61)
Stage IV + V	168	(4.7)	528	(3.8)	49	(3.9)	137	(3.6)	882	(3.9)
**Ipsilateral degree of stenosis**										
Mild (< 50%, NASCET)	81	(2.3)	215	(1.6)	16	(1.3)	45	(1.2)	357	(1.6)
Moderate (50–69%, NASCET)	231	(6.5)	779	(5.6)	38	(3.0)	166	(4.4)	1214	(5.4)
Severe (70–99%, NASCET)	3106	(87)	12,574	(91)	1201	(95)	3519	(94)	20,400	(91)
Occlusion (100%)	147	(4.1)	297	(2.1)	7	(0.6)	24	(0.6)	475	(2.1)
**Contralateral degree of stenosis**										
Mild (< 50%, NASCET)	2647	(74)	9621	(69)	894	(71)	2511	(67)	15,673	(70)
Moderate (50–69%, NASCET)	382	(11)	1966	(14)	191	(15)	548	(15)	3087	(14)
Severe (70–99%, NASCET)	321	(9.0)	1495	(11)	117	(9.3)	503	(13)	2436	(11)
Occlusion (100%)	215	(6.0)	783	(5.6)	60	(4.8)	192	(5.1)	1250	(5.5)
**Indication Group**										
- Group A (asymptomatic)	1753	(49)	7274	(52)	842	(67)	2444	(65)	12,313	(55)
- Group B (symptomatic, elective)	1265	(35)	5013	(36)	352	(28)	991	(26)	7621	(34)
AFX or TIA*	*622*	*(49)*	*2284*	*(46)*	*152*	*(43)*	*489*	*(49)*	*3547*	*(47)*
Stroke*	*594*	*(47)*	*2507*	*(50)*	*177*	*(50)*	*438*	*(44)*	*3716*	*(49)*
Other symptoms*	*49*	*(4)*	*222*	*(4)*	*23*	*(7)*	*64*	*(6)*	*358*	*(4.7)*
- Group C (others)	547	(15)	1578	(11)	68	(5.4)	319	(8.5)	2512	(11)
Crescendo-TIA/Stroke-in-evolution*	*234*	*(43)*	*747*	*(47)*	*27*	*(40)*	*117*	*(37)*	*1125*	*(45)*
*Simultaneous procedures*^#^*	*185*	*(34)*	*294*	*(19)*	*10*	*(15)*	*87*	*(27)*	*576*	*(23)*
Others°*	*128*	*(23)*	*537*	*(34)*	*31*	*(46)*	*115*	*(36)*	*811*	*(32)*

If not stated otherwise, percentages refer to the column. Q1 = first quartile, Q3 = third quartile, ASA = American Society of Anaesthesiologists physical status classification system. * = percentages refer to the respective subgroup, # = simultaneous performed coronary bypass operation, peripheral arterial reconstruction, aortic procedure, intracranial stenting, and other simultaneous performed procedures. ° = carotid aneurysm, symptomatic coiling, exulcerated plaque morphology, ipsilateral carotid occlusion, redo carotid procedures, tandem stenosis. $ = 294 patients with unknown ASA stage were excluded

### Characteristics of treating hospitals and regional conditions

On a hospital level, 95% of patients were treated in centres reporting on-site vascular surgery experience (Table 2). Treating departments had a certification of the German Vascular Society (DGG) in 33% of cases. A certified stroke unit (SU, German Stroke Society) was available on-site in 33% (supra-regional SU), 20% (regional SU), and 2% (telemedicine stroke network). Most patients were treated in centres located in independent cities (47%), followed by hospitals in sparsely populated regions (26%), and rural districts (19%). For further regional characteristics of the treating hospitals, please see Supplemental Table 1. University hospitals had higher median annual caseloads of CEA (90 per year) and CAS (39 per year), compared to private hospitals (37 and 1, respectively).

**Table 2:** Characteristics of patients regarding hospital characteristics

Unit of analysis = patient	Hospital ownership								all patients	
	University		Public		Charitable		Private			
**Specialists available at centre** ^$^										
Vascular surgeon	3565	(100)	13,008	(94)	1247	(99)	3542	(94)	21,362	(95)
Heart surgeon	2514	(71)	5193	(37)	542	(43)	1342	(36)	9591	(43)
Angiologist	1736	(49)	6825	(49)	876	(69)	2104	(56)	11,541	(51)
Cardiologist	3565	(100)	13,127	(95)	1259	(100)	3186	(85)	21,137	(94)
Neurologist	3363	(94)	10,190	(73)	957	(76)	2550	(68)	17,060	(76)
Neurosurgeon	3565	(100)	9409	(68)	735	(58)	1499	(40)	15,208	(68)
Neuroradiologist	3565	(100)	5675	(41)	658	(52)	1251	(33)	11,149	(50)
**On-site certified vascular centre** ^**&**^										
DGG	1804	(51)	4343	(31)	542	(43)	611	(16)	7300	(33)
DGA	0	(0)	443	(3.2)	542	(43)	0	(0)	985	(4.4)
DRG	217	(6.1)	2671	(19)	513	(41)	0	(0)	3401	(15)
**Certified Stroke-unit (DSG) on-site**										
Supra-regional	3029	(85)	3548	(26)	542	(43)	252	(6.7)	7371	(33)
Regional	0	(0)	2895	(21)	0	(0)	1519	(40)	4414	(20)
Telemedicine stroke network	0	(0)	353	(2.5)	0	(0)	36	(1.0)	389	(1.7)
None	536	(15)	7069	(51)	720	(57)	1947	(52)	10,272	(46)
**Certified Quality management system**										
DIN ISO EN 9000	1327	(37)	1985	(14)	611	(48)	489	(13)	4412	(20)
KTQ	149	(4)	1362	(10)	29	(2)	0	(0)	1540	(6.9)
proCum Cert	0	(0)	0	(0)	384	(30)	0	(0)	384	(1.7)
none of these	2089	(59)	10,518	(76)	238	(19)	3265	(87)	16,110	(72)
**Centre annual caseload (median; Q1–Q3)**										
CEA	90	(79–141)	20	(4–49)	19	(2–31)	37	(13–65)	24	(5–55)
CAS	39	(17–57)	1	(0–8)	0	(0–5)	1	(0–6)	2	(0–9)
**Regional settlement structure**										
Independent city	3565	(100)	5087	(37)	936	(74)	922	(25)	10,510	(47)
Urban district	0	(0)	891	(6.4)	0	(0)	837	(22)	1728	(8)
Rural district	0	(0)	3635	(26)	0	(0)	689	(18)	4324	(19)
Sparsely populated region	0	(0)	4252	(31)	326	(26)	1306	(35)	5884	(26)

DGG = German Vascular Society; DGA = German Society for Angiology; DRG = German Society for Radiology; DSG = German Stroke Society; CEA = carotid endarterectomy, CAS = carotid artery stenting. n = patients with feature or property, N = all patients with information available, Q1 = first quartile, Q3 = third quartile. & = each percentage refer to the total number per column. Double or triple count possible, therefore the sum does not have to add up to 100%. $ = as specified in the annual quality report

### Management and treatment of patients

CEA was the preferred revascularization method for asymptomatic (85%) and symptomatic patients (84%) respectively (Table 3). Preprocedural assessment by a neurologist was performed in 65% of patients, and after the procedure in 57% (Table 3). Both, pre- and postprocedural assessment by a neurologist was carried out in 49%, and was more frequent in university hospitals compared to private hospitals (52% vs. 31%). For CEA, local and general anaesthesia were carried out in 25% and 71%, respectively. For further details on management and treatment, see Table 3. The share of symptomatic patients treated within 1 week after index event was 42% in private hospital and 58% in non-private hospitals. In turn, the proportion of symptomatic patients treated longer than two weeks after index event was 39% in private hospitals, and 24% in non-private centres.

**Table 3:** Diagnostic procedures, management, and treatment of patients

	Hospital ownership								all patients	
	University		Public		Charitable		Private			
Time interval* (median, Q1–3)	7	(4-14)	6	(4-12)	7	(4-15)	9	(5-20)	n.a.	
0–2 days	161	(15)	643	(15)	39	(13)	76	(9)	919	(14)
3–7 days	457	(42)	1834	(44)	109	(37)	262	(32)	2662	(42)
8–14 days	169	(16)	778	(19)	47	(16)	157	(19)	1151	(18)
15–180 days	294	(27)	915	(22)	98	(33)	314	(39)	1621	(26)
**Assessment by a neurologist**										
Preprocedural	2637	(74)	9579	(69)	741	(59)	1696	(45)	14,653	(65)
Postprocedural	1931	(54)	7962	(57)	1070	(85)	1854	(49)	12,817	(57)
Pre- and postprocedural	1871	(52)	7334	(53)	676	(54)	1167	(31)	11,048	(49)
**Perioperative antiplatelet medication**										
Mono therapy^#^	2709	(76)	11,398	(82)	1141	(90)	2894	(77)	18,142	(81)
Dual antiplatelet medication	630	(18)	1971	(14)	92	(7)	249	(7)	2942	(13)
None	226	(6)	496	(4)	29	(2)	611	(16)	1362	(6.1)
**Treatment by indication group**										
- Group A (asymptomatic)										
CEA	1416	(81)	6074	(84)	761	(90)	2269	(93)	10,520	(85)
CAS	337	(19)	1200	(16)	81	(10)	175	(7.2)	1793	(15)
- Group B (symptomatic, elective)										
CEA	987	(78)	4246	(85)	307	(87)	882	(89)	6422	(84)
CAS	278	(22)	767	(15)	45	(13)	109	(11)	1199	(16)
- Group C (others)										
CEA	253	(46)	994	(63)	49	(72)	255	(80)	1551	(62)
CAS	294	(54)	584	(37)	19	(28)	64	(20)	961	(38)
**Type of anaesthesia (only CEA)** ^§^										
Local anaesthesia	539	(33)	1488	(22)	137	(18)	669	(33)	2833	(25)
General anaesthesia	1054	(65)	5042	(73)	610	(81)	1314	(65)	8020	(71)
Combined anaesthesia	19	(1.2)	375	(5.4)	7	(0.9)	26	(1.3)	427	(3.8)
**Intraprocedural monitoring** ^§§^										
Electroencephalography	0	(0)	226	(2.7)	0	(0)	25	(1.1)	251	(1.9)
Transcranial Cerebral Oximetry	248	(12)	846	(10)	124	(15)	73	(3.3)	1291	(9.5)
Somato-sensory evoked potentials	351	(17)	1504	(18)	1	(0.1)	676	(31)	2532	(19)
Other methods	646	(31)	1539	(18)	88	(11)	331	(15)	2604	(19)

Q1 = first quartile, Q3 = third quartile; * = Time interval between the index event and time of treatment (only for indication group B), # = ASS, Clopidogrel, other antiplatelet medication, § = available only from 2014–2016, *n* = 11,280, §§ = available only from 2014–2016, *n* = 13,504, (column percent refer to the respective size of sub-cohort)

### Outcome of treatment

**Fig. 2:**
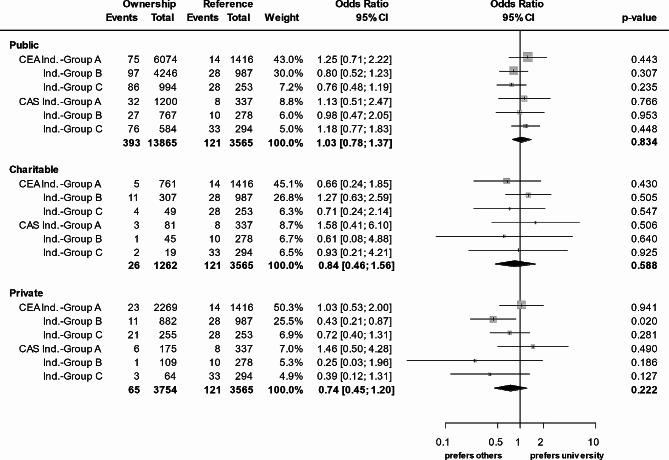
Raw odds ratio for any in-hospital stroke or death (primary outcome event) by hospital ownership each compared to university hospitals (reference). Univariate analysis CEA = carotid endarterectomy, CAS = carotid artery stenting, CI = confidence interval, Ind.-Group A = asymptomatic, Ind.-Group B = symptomatic elective, Ind.-Group C = symptomatic emergency, crescendo-TIA, stroke-in-evolution, other indications

Overall risk of stroke or death in asymptomatic patients was 1,1% in patients treated with CEA, and 2,7% in those who underwent CAS (Table 4). In symptomatic patients (elective treatment), the risks were 2,3% and 3,3%, respectively. In asymptomatic patients, the risk of stroke or death was 1,3% in university hospitals, 1,5% in public hospitals, 1,0% in hospitals of charitable owners, and 1,2% in private hospitals. In symptomatic patients, these figures were 3,0%, 2,5%, 3,4%, and 1,2% respectively. Univariate analysis revealed no statistically significant differences between hospital groups (Fig. 2). Only the subgroup of patients treated electively for symptomatic carotid stenosis (indication group B) in private hospitals showed a significantly lower risk for any stroke or death (0.43 [95%-CI 0.21–0.87, *p* = 0.020]).

**Table 4:** Primary outcomes of treatment

	Hospital ownership								all patients	
	University		Public		Charitable		Private			
**Carotid endarterectomy (CEA)**										
**Any stroke or death**										
- Group A: asymptomatic	14/1416	(1.0)	75/6074	(1.2)	5/761	(0.7)	23/2269	(1.0)	117/10,520	(1.1)
- Group B: symptomatic, elective	28/987	(2.8)	97/4246	(2.3)	11/307	(3.6)	11/882	(1.2)	147/6422	(2.3)
- Group C1: symptomatic, emergency*	16/107	(15.0)	40/482	(8.3)	3/22	(13.6)	10/106	(9.4)	69/717	(9.6)
- Group C2: Simultaneous procedures^#^	6/70	(8.6)	14/129	(10.9)	0/4	(0.0)	6/55	(10.9)	26/258	(10.1)
- Group C3: Others°	6/76	(7.9)	32/383	(8.4)	1/23	(4.3)	5/94	(5.3)	44/576	(7.6)
**Carotid artery stenting (CAS)**										
**Any stroke or death**										
- Group A: asymptomatic	8/337	(2.4)	32/1200	(2.6)	3/81	(3.7)	6/175	(3.4)	49/1793	(2.7)
- Group B: symptomatic, elective	10/278	(3.6)	27/767	(3.5)	1/45	(2.2)	1/109	(0.9)	39/1199	(3.3)
- Group C1: symptomatic, emergency*	15/127	(11.8)	34/265	(12.8)	1/5	(20.0)	1/11	(9.1)	51/408	(12.5)
- Group C2: Simultaneous procedures^#^	12/115	(10.4)	22/165	(13.3)	1/6	(16.6)	0/32	(0.0)	35/318	(11.0)
- Group C3: Others°	6/52	(11.5)	20/154	(13.0)	0/8	(0.0)	2/21	(9.5)	28/235	(11.9)

Q1 = first quartile, Q3 = third quartile; * = subgroup of group C suffering from Crescendo-TIA or Stroke-in-evolution; # = simultaneous performed coronary bypass operation, peripheral arterial reconstruction, aortic procedure, intracranial stenting, and other simultaneous performed procedures; ° = carotid aneurysm, symptomatic coiling, exulcerated plaque morphology, ipsilateral carotid occlusion, redo carotid procedures, tandem stenosis

**Fig. 3:**
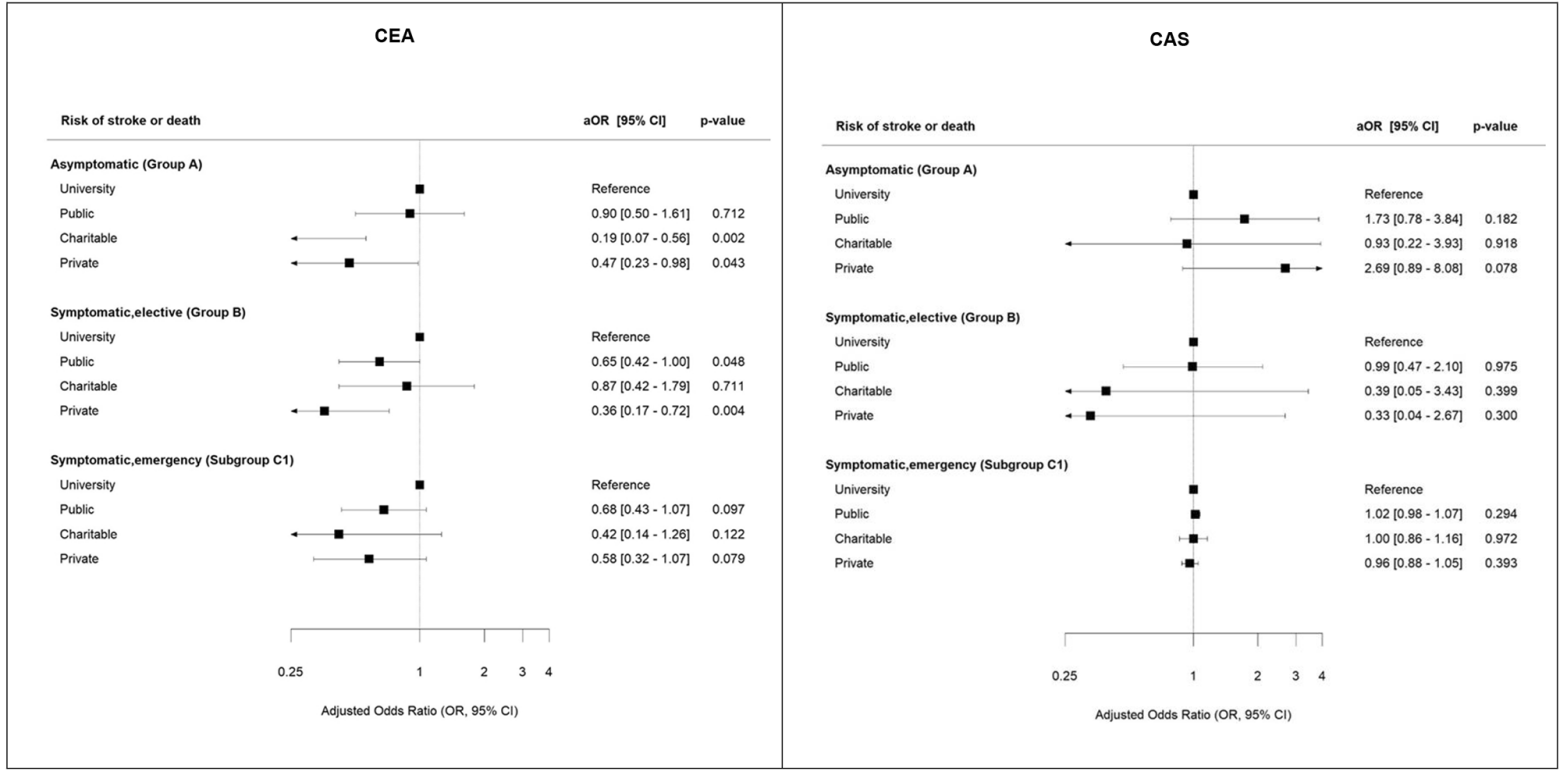
Multivariable regression analysis for patients treated with CEA (left) and CAS (right), basic models, adjusted only for pre- and post-procedural examination by a neurologist aOR = odds ratio adjusted for pre- and post-procedural examination by an neurologist, CI = confidence interval

In the multivariable regression analysis (basic model, Fig. 3), compared to university hospitals, the odds ratio (OR) of stroke or death in asymptomatic patients treated with CEA was significantly lower in charitable hospitals (0.19 [95%-CI 0.07–0.56, *p* = 0.002]) and private hospitals (0.47 [95%-CI 0.23–0.98, *p* = 0.043]). In symptomatic patients (elective treatment, CEA), patients treated in private or public hospitals showed a significantly lower OR compared to university hospitals (0.36 [95%-CI 0.17–0.72, *p* = 0.004] and 0.65 [95%-CI 0.42–1.00, *p* = 0.048], respectively).

The extended multivariable regression model showed formally better fitting parameter compared to basic model (Supplemental Table 2), but no qualitative differences to the simpler models (Supplemental Fig. 1). For secondary outcomes, please see Supplemental Table 3 for CEA, and Supplemental Table 4 for CAS.

## Discussion

This is the first analysis of the interrelationship between hospital ownership and patient selection in relation to patient selection, treatment and outcome of carotid endarterectomy (CEA) and carotid artery stenting (CAS) in Germany.

In summary, our data indicate that Bavarian private hospitals (as compared to non-private hospitals) predominantly treat asymptomatic elderly and multimorbid patients, have a lower annual case load, use more frequently CEA as compared to CAS, are less often certified as vascular centres and/or stroke units, treat symptomatic patients later, and perform specialist neurological assessments less frequently. It is also striking that despite the German-Austrian guideline published in 2012, a high number of asymptomatic patients continued to be treated, 15% of them with CAS. In addition, it is noticeable that the annual case numbers for CAS are well below the current recommendations for minimum volumes, except for university hospitals. However, it must be borne in mind that although the annual number of cases can serve as a surrogate marker for the skills of the treating centre or physician, methodological aspects such as device selection, the technical development of stents and the use of protection systems can also have an influence on the interventional result and outcomes. Additionally, a pre- and post-procedural specialist neurological examination is also not routinely performed, especially not in private clinics. These aspects could serve as a starting point for public health measures to improve guideline-adherence in Germany. In total, 17% of carotid revascularization procedures (CEA, CAS) were performed in private hospitals, which is comparable to other countries, e.g. USA 14–18% [14]. As about 19% of all hospital beds in Germany were for-profit, these shares match well [3].

The proportion of asymptomatic patients (indication group A) comprises about 50% in university and public hospitals, and about 66% in charitable and private hospitals. These values appear very high, but are within the international range of reported rates, e.g., 92% for the USA, 15% for the United Kingdom, and 0–79% for Australia [14, 32, 33]. Among other things, these rates may be influenced by the remuneration system and the medical guidelines applied. Regarding this, in Germany, national and international guidelines [30, 34] are the accepted standard regarding diagnosis, indication for treatment, procedure selection, and follow-up of carotid artery stenosis.

The median age of patients was lowest in university hospitals (71 years), and highest in public and private hospitals (2 years higher). An older age of patients treated in private clinics was also reported for CEA/CAS and implantation of left ventricular assist devices (LVAD) performed in the USA (Chandler et al. [14], Briasoulis et al. [11], data from the Nationwide Inpatient Sample, NIS).

The proportion of sicker patients with ASA III–V is largest in private hospitals (76%) and smallest in university hospitals (62%). This seems counterintuitive, as it is generally assumed that more severely ill patients are treated in university hospitals. It remains to be seen whether this is actually the case or whether different assessment standards are applied in the evaluation of ASA category leading to a misclassification bias. In Germany, surgically treated patients have higher ASA stages than patients receiving CAS, which seems also counterintuitive. However, it must be borne in mind that the ASA classification for CEA is presumably carried out by anaesthesiologists as part of the preoperative preparation. With regard to CAS, however, it is conceivable that these are routinely performed with anaesthesia standby, or not, and then the classification is performed by radiologists or other interventional physicians who may systematically misclassify the ASA stage. Since private clinics perform more CEA than CAS, the differences could be purely due to the different assessment of CEA and CAS patients in terms of ASA stage. However, no differences regarding comorbidity of CEA/CAS patients were found for the USA [14] or for patients receiving LVAD [11].

In symptomatic patients (indication group B), the time interval between the index event and therapy was longer on average in private hospitals than in non-private hospitals. The proportion of urgent treatment (0–2 days of the index event) was nearly twice as high in nonprivate hospitals as in private hospitals. In contrast, the proportion of patients who underwent delayed surgery (> 14 days after index event) was just half as high in non-private facilities as in private centres. Overall, in contrast to the USA or Australia [33, 35], CEA and CAS were performed with comparable delay. The better outcome rates for symptomatic patients (treated electively, indication group B) in private clinics may be due to the presumption that patients in this subcohort who require more “urgent elective” treatment are nevertheless treated in non-private clinics.

The proportion of patients treated endovascularly (CAS) is largest in university hospitals (25%) and smallest in private hospitals (9%). On average, this rate is comparable to results from the USA (17.4% [14]). Delayed timing of treatment was associated with lower rate of stroke and death in Germany (by trend) [24, 25] and the USA (significantly) [35], with fundamentally higher risks for CAS compared to CEA. Therefore, as depicted in Fig. 2, the amazingly low raw risk of stroke or death in private hospitals in symptomatic patients (indication group B) might be due to treatment timing and avoiding the riskier endovascular treatment. However, proper patient selection – either active or passive – might also have caused this observation. Except for the indication group B, no different raw outcome risks for any in-hospital stroke or death were found with regard to hospital ownership in the univariate analysis. However, after multivariable adjustment, the risk of stroke or death is significantly lower in private hospitals in all non-emergency patients (indication groups A and B). In addition, the adjusted risk of stroke or death is lower in public hospitals (indication group B), and charitable hospitals (indication group A).

This study shows that the patient cohorts differ depending on the hospital ownership (possible provider-induced effect) and that the outcomes are also different (possible difference in quality). Despite extensive adjustments, it cannot be ruled out that differences in patient structure and not just local medical care influenced the outcomes.

Except for slight quantitative differences, inflating of the regression model by integration of further variables in addition to neurological assessments did not lead to qualitative changes in the associations. Although regression models were specified a-priori, model selection may have influenced results, which can be seen when comparing raw data with multiple adjusted regression analysis. However, according to the principle of parsimony (or Ockham’s razor), the simpler model (basic model) was finally preferred. This model only adjusts for pre- and postprocedural assessment by a neurologist, which were the strongest confounders in all previous analyses of the German nationwide carotid quality assurance database [17–26]. As a procedural factor, the pre-procedural specialist neurological examination can certainly be classified as a confounder. In contrast, the post-procedural specialist neurological examination should be viewed in a more differentiated way. On the one hand, it can be regarded as a confounder that increases the probability of measuring the outcome more precisely, but on the other hand it can also be a surrogate marker for suspected or occurring post-procedural abnormalities. Since the reason for performing the post-procedural specialist neurological examination is not documented (e.g. by routine, by random or only because of suspected stroke), a certain bias due to confounding-by-indication cannot be ruled out. This should be taken into account when interpreting the univariate and basic model. Nevertheless, for transparency reasons, also the results from the extended model are presented.

As effect estimates diverge from 1.0 (null effect) when adjusting for pre- and postprocedural assessment by a neurologist, these variables may also be considered ‘negative’ confounders [36]. In former analyses of our group, *pre*-procedural neurological assessment was significantly associated with lower outcome risks, while *post*-procedural examination was significantly associated to higher outcome risks (data not separately published). A detailed analysis of these factors is the subject of a further predefined analysis of the ISAR-IQ project (not yet published). The absolute value of effect estimate for pre-procedural examination was always smaller than that of post-procedural examination. Because it is not documented whether the postprocedural examination was routine or only due to a suspected neurologic deficit (diagnostic suspicion bias), this variable itself may be subject to confounding by indication. For example, it seems conclusive that more deficits are detected by more frequent routine postprocedural neurological examination. In turn, it is also conceivable that if a postprocedural deficit leads more frequently to a neurologic examination, the variable ‘neurological examination’ itself is now associated with outcome and thus, becomes a ‘risk’ factor. When adjusting for this risk factor in multivariable regression analysis, the OR for outcome decreases in centres that frequently perform post-procedural neurological control routinely, although the raw risk remains unchanged. Post-procedural assessment by a neurologist was most frequently performed in charitable hospitals (85%) and public centres (57%). In turn, pre-procedural assessment by a neurologist was most frequently performed in university hospitals (74%) and least frequently in private centres (45%). Therefore, the authors consider it possible that the lower risk of stroke or death found only in the multivariable analyses may be biased by the rate of preprocedural and postprocedural neurologic examinations (information bias).

Nevertheless, a clear cause of the better performance in private hospitals cannot be proven on the basis of the available data. However, if you hypothetically put yourself in the situation of a CEO or middle manager of a profit-oriented company, I would do everything to make the value chain as efficient and effective as possible. For the treatment of carotid stenosis, for example, I would thus hire the best physicians, nurses and functional staff, provide them with the optimal technical equipment and optimize the intra-clinical processes to the hilt. Good structural and process quality probably leads to good outcome quality and thus fewer costly complications, which in turn leads to more revenue for hospitals in the DRG-System and more return for investors. Although these speculations cannot be proven by existing data, they seem obvious from a business perspective. Nevertheless, it is not possible to assess whether the business impetus for such optimization measures is comparable in non-private hospitals.

### Limitations

This is a secondary data analysis and thus, all shortcomings of observational studies using routine data must be considered in principle. These limitations were discussed in detail elsewhere [17–26, 29, 37] and will be summarized here:

- First, the study design was retrospective and observational.
- Second, follow-up data covered only the inhospital period.
- Third, all data in the database are self-reported by the attending physicians, and reporting bias cannot be ruled out.
- Fourth, ASA stage was the only variable besides age, sex, degree of stenosis, symptom status and others that could be used for risk adjustment. Additionally, unobserved confounders as well as selection bias caused by patient choice or regional differences (e.g. driving distances to the next hospital) may have influenced the results [38].
- Fifth, for data protection reasons, the unique hospital identifier is pseudonymised when the data are transmitted to the IQTIG, so that linking the level 1 data with those of levels 2 and 3 was unfortunately only possible for Bavaria.
- Last, unfortunately, the revenue for individual patients is not documented in the clinical quality assurance data.

You will find a detailed discussion of the limitations in the supplement.

## Conclusion

This study shows that patient selection and treatment were related to hospital ownership. No general association between hospital ownership and outcome of treatment was found. The lower risk of stroke or death in the subgroup of electively treated patients in private hospitals might be due to the right timing or the choice of treatment modality. However, it might also be due to the stringently optimized structural and process quality in a profit-oriented operating environment in private hospitals. Nonetheless, residual information bias or confounding by indication must be considered when interpreting the results.

### Electronic supplementary material

Below is the link to the electronic supplementary material.

Supplementary Material 1

## Data Availability

The datasets analysed during the current study are available on request from the IQTIG, (https://iqtig.org/qs-verfahren-uebersicht/sekundaere-datennutzung/). All protocols were approved by the IQTIG (Institut für Qualitätssicherung und Transparenz im Gesundheitswesen) and the G-BA (Germany’s Federal Joint Committee). In accordance with § 137a (10) of Book V of the German Social Code (SGB V), the IQTIG makes all data collected during the mandatory quality assurance measures in accordance with § 136 (1) sentence 1 no. 1 SGB V available for secondary scientific purposes. The Federal Joint Committee (G-BA) has defined regulations for a transparent procedure for the use of this data in accordance with § 137a (10) sentence 4 SGB V in its Code of Procedure (VerfO) Chapter 8.

## Abbreviations

AIC: Akaike information criterion
ASA: American Society of Anesthesiologists
BAQ: Bayerische Arbeitsgemeinschaft Qualitätssicherung
CAS: Carotid artery stenting
CEA: Carotid endarterectomy
CI: Confidence interval
DGG: German Vascular Society
DRG: Diagnosis related group
G-BA: Germany’s Federal Joint Committee (Gemeinsamer Bundesausschuss)
INKAR: Indikatoren und Karten zur Raum- und Stadtentwicklung
IQTIG: Institute for Quality Assurance and Transparency in Healthcare
MACE: Major adverse cardiovascular event
NUTS: Nomenclature des Unités territoriales statistiques
OR: Odds ratio
SGB: German social security code (law)
