# Supplementary material for: Association between hospital ownership and patient selection, management, and outcomes after carotid endarterectomy or carotid artery stenting: – Secondary data analysis of the Bavarian statutory quality assurance database –
Source: BMC Surg. 2024 May 17;24:158. doi: 10.1186/s12893-024-02448-6 (PMC11100040; doi:10.1186/s12893-024-02448-6)
Supplement: Supplementary file 1 — Supplementary Material 1 [file 12893_2024_2448_MOESM1_ESM.pdf]

Supplemental Tables:

Supplemental Table 1: Regional characteristics of the counties (NUTS Level 3) of the treating hospitals.								
	**Hospital ownership**							
	**University**		**Public**		**Charitable**		**Private**	
**Socioeconomic indices**								
UX (M; Q1–3)	1.18	(0.99-3.95)	0.38	(-0.16-0.92)	1.84	(1.15-4.03)	0.27	(-0.86-3.89)
SGX (M; Q1–3)	-0.92	(-1.08- -0.58)	-0.79	(-1.24 - -0.23)	-0.88	(-1.15- -0.02)	-1.08	(-1.47- -0.40)
GISD (M; Q1–3)	0.10	(0.26-0.21)	0.35	(0.30-0.40)	0.14	(0.05-0.36)	0.32	(0.10-0.40)
**Regional outpatient care (headcount)**								
Vascular surgeons	8	(3-15)	3	(3-3)	9	(3-16)	3	(0-14)
Angiologists / Cardiologists	12	(8-107)	3	(3-8)	30	(9-107)	3	(3-106)
Neurologists	14	(6-75)	3	(3-6)	27	(13-77)	3	(3-73)
General Practitioners	119	(112-1139)	79	(54-106)	381	(113-1144)	99	(61-1116)
**Accessibility (by car, in minutes)**								
medium-size municipality	0	(0-0)	5	(0-8)	0	(0-0)	7	(0-9)
large-size city	0	(0-0)	11	(0-17)	0	(0-0)	18	(0-25)
**Health care structures (density#)**								
General Practitioners*	72	(72-74)	67	(62-74)	72	(70-74)	72	(69-73)
Hospitals	3.43	(3.15-3.53)	3.43	(2.30-4.73)	3.31	(2.42-3.44)	3.49	(3.17-6.27)
Hospital beds	1876	(794-1932)	687	(354-1211)	783	(767-945)	783	(506-1007)
Hospital beds vascular surgery	6.93	(6.01-22.68)	0	(0-23.04)	6.74	(6.01-13.47)	6.93	(0-39.15)
Hospital beds neurology	84.49	(39.06-133.37)	32.55	(0-71.90)	36.25	(19.22-39.06)	39.06	(32.21-82.93)
Stroke units	0.91	(0.35-1.37)	0.35	(0-1.35)	0.34	(0.20-0.35)	0.35	(0.08-0.74)
DGG-certified vascular centres	0.21	(0-0.80)	0	(0-0)	0.21	(0-0.21)	0	(0-0.20)

NUTS = Nomenclature des unités territoriales statistiques. UX = urbanity index (Urbanitätsindex), SGX = socioeconomic health index (Sozioökonomischer Gesundheitsindex), GISD = German Index of Socioeconomic Deprivation. DGG = German Vascular Society. M = Median, Q1 = first quartile, Q3 = third quartile. * = available only from 2015–2017. # = per 100.000 inhabitants.

Supplemental Table 2: Akaike information criterion (AIC) for the three different regression models (lower is better, a Δ_{AIC} of >2 is considered 'substantial' [31]).				
**Treatment**	**Indication Group**	**Univariate model**	**Basic model**	**Extended model**
CEA	A	1290.50	1201.00	1187.20
	B	1400.70	1372.50	1077.70
	C	942.13	899.64	858.81
CAS	A	456.69	420.58	393.29
	B	348.86	336.23	271.88
	C	563.17	560.54	666.85

Univariate model: no adjustment
Basic model: adjustment for pre- and post-procedural assessment by a neurologist
Extended model: adjustment for age, sex, ASA, ipsi- and contralateral degree of stenosis, pre- and post-procedural assessment by neurologist, and annual caseload of treating centre.
For indication group B, additional adjustment for neurological symptoms and time interval between index event and treatment.

Supplemental Table 3: Secondary outcomes in patients treated with carotid endarterectomy (CEA).								
**Carotid endarterectomy (CEA)**	**Hospital ownership**							
	**University**		**Public**		**Charitable**		**Private**	
**Group A: asymptomatic**								
Post-OP Hospital stay in days (M; Q1–Q3)	3	(3-4)	4	(3-5)	4	(4-5)	4	(4-5)
Major stroke or death	8	(0.6)	50	(0.8)	4	(0.5)	14	(0.6)
MI	4	(0.5)	10	(0.3)	1	(0.2)	1	(0.1)
Any stroke	12	(0.8)	62	(1.0)	3	(0.4)	19	(0.8)
All-cause death	2	(0.1)	19	(0.3)	2	(0.3)	4	(0.2)
**Group B: symptomatic, elective**								
Post-OP Hospital stay in days (M; Q1–Q3)	4	(3-5)	5	(4-6)	5	(4-7)	5	(4-6)
Major stroke or death	20	(2.0)	77	(1.8)	11	(3.6)	10	(1.1)
MI	7	(1.2)	4	(0.2)	0	(0.0)	0	(0.0)
Any stroke	20	(2.0)	71	(1.7)	7	(2.3)	11	(1.2)
All-cause death	10	(1.0)	33	(0.8)	6	(2.0)	0	0
**Group C1: symptomatic**								
Post-OP Hospital stay in days (M; Q1–Q3)	5	(3-8)	5	(4-8)	7	(5-13)	6	(4-9)
Major stroke or death	14	(13)	36	(7.5)	3	(14)	6	(5.7)
MI	0	(0.0)	3	(0.9)	0	(0.0)	0	(0.0)
Any stroke	12	(11)	31	(6.4)	2	(9.1)	8	(7.5)
All-cause death	6	(5.6)	14	(2.9)	1	(4.5)	2	(1.9)
**Group C2: emergency***								
Post-OP Hospital stay in days (M; Q1–Q3)	9	(6-13)	10	(6-14)	5	(5-5)	12	(7-20)
Major stroke or death	5	(7.1)	13	(10)	0	(0.0)	6	(11)
MI	0	(0.0)	0	(0.0)	0	(0.0)	1	(2.9)
Any stroke	1	(1.4)	5	(3.9)	0	(0.0)	3	(5.5)
All-cause death	5	(7.1)	11	(8.5)	0	(0.0)	4	(7.3)
**Group C3: other patients**								
Post-OP Hospital stay in days (M; Q1–Q3)	4	(3-5)	5	(4-7)	5	(4-6)	4	(4-6)
Major stroke or death	4	(5.3)	29	(7.6)	0	(0.0)	3	(3.2)
MI	0	(0.0)	3	(1.2)	0	(0.0)	0	(0.0)
Any stroke	4	(5.3)	21	(5.5)	1	(4.3)	4	(4.3)
All-cause death	3	(3.9)	15	(3.9)	0	(0.0)	1	(1.1)

M = Median, Q1 = first quartile, Q3 = third quartile, * = subgroup of group C suffering from Crescendo-TIA or Stroke-in-evolution. MI = myocardial infarction (available only from 2012 to 2016).

Supplemental Table 4: Secondary outcomes in patients treated with carotid artery stenting (CAS).								
**Carotid artery stenting (CAS)**	**Hospital ownership**							
	**University**		**Public**		**Charitable**		**Private**	
**Group A: asymptomatic**								
Post-OP Hospital stay in days (M; Q1–Q3)	3	(2-3)	2	(1-3)	2	(2-3)	2	(2-2)
Major stroke or death	5	(1.5)	20	(1.7)	2	(2.5)	5	(2.9)
MI	0	(0.0)	0	(0.0)	0	(0.0)	0	(0.0)
Any stroke	8	(2.4)	29	(2.4)	2	(2.5)	6	(3.4)
All-cause death	0	(0.0)	7	(0.6)	1	(1.2)	1	(0.6)
**Group B: symptomatic, elective**								
Post-OP Hospital stay in days (M; Q1–Q3)	3	(2-6)	3	(2-6)	3	(2-5)	2	(2-4)
Major stroke or death	7	(2.5)	19	(2.5)	1	(2.2)	0	(0.0)
MI	1	(0.7)	0	(0.0)	0	(0.0)	0	(0.0)
Any stroke	8	(2.9)	20	(2.6)	1	(2.2)	1	(0.9)
All-cause death	4	(1.4)	8	(1.0)	0	(0.0)	0	(0.0)
**Group C1: symptomatic**								
Post-OP Hospital stay in days (M; Q1–Q3)	8	(5-13)	8	(5-13)	10	(8-10)	10	(7-15)
Major stroke or death	13	(10)	34	(13)	1	(0)	1	(9.1)
MI	1	(1.8)	0	(0.0)	0	(0.0)	0	(0.0)
Any stroke	7	(5.5)	13	(4.9)	1	(20)	1	(9.1)
All-cause death	10	(7.9)	27	(10)	0	(0.0)	0	(0.0)
**Group C2: emergency***								
Post-OP Hospital stay in days (M; Q1–Q3)	9	(4-13)	8	(3-14)	4	(3-13)	3	(3-7)
Major stroke or death	10	(8.7)	21	(13)	1	(17)	0	(0.0)
MI	0	(0.0)	0	(0.0)	0	(0.0)	0	(0.0)
Any stroke	5	(4.3)	13	(7.9)	1	(17)	0	(0.0)
All-cause death	8	(7.0)	19	(12)	0	(0.0)	0	(0.0)
**Group C3: other patients**								
Post-OP Hospital stay in days (M; Q1–Q3)	6	(3-12)	6	(3-12)	4	(2-10)	2	(2-5)
Major stroke or death	4	(7.7)	15	(9.7)	0	(0.0)	1	(4.8)
MI	0	(0.0)	0	(0.0)	0	(0.0)	0	(0.0)
Any stroke	3	(5.8)	5	(5.1)	0	(0.0)	1	(4.8)
All-cause death	4	(7.7)	12	(7.8)	0	(0.0)	1	(4.8)

M = Median, Q1 = first quartile, Q3 = third quartile, * = subgroup of group C/D suffering from Crescendo-TIA or Stroke-in-evolution. MI = myocardial infarction (available only from 2012 to 2016).

Supplemental figures:

Supplemental Figure 1: Multivariable regression analysis (extended model) for patients treated with CEA (left) and CAS (right).

CEA

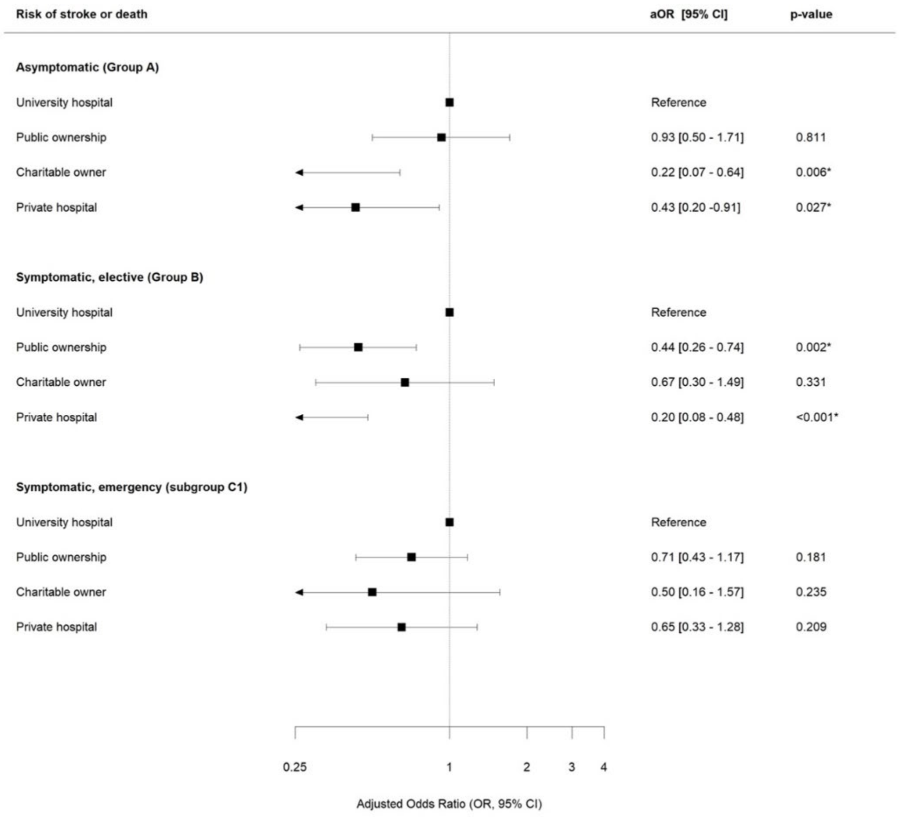

CAS

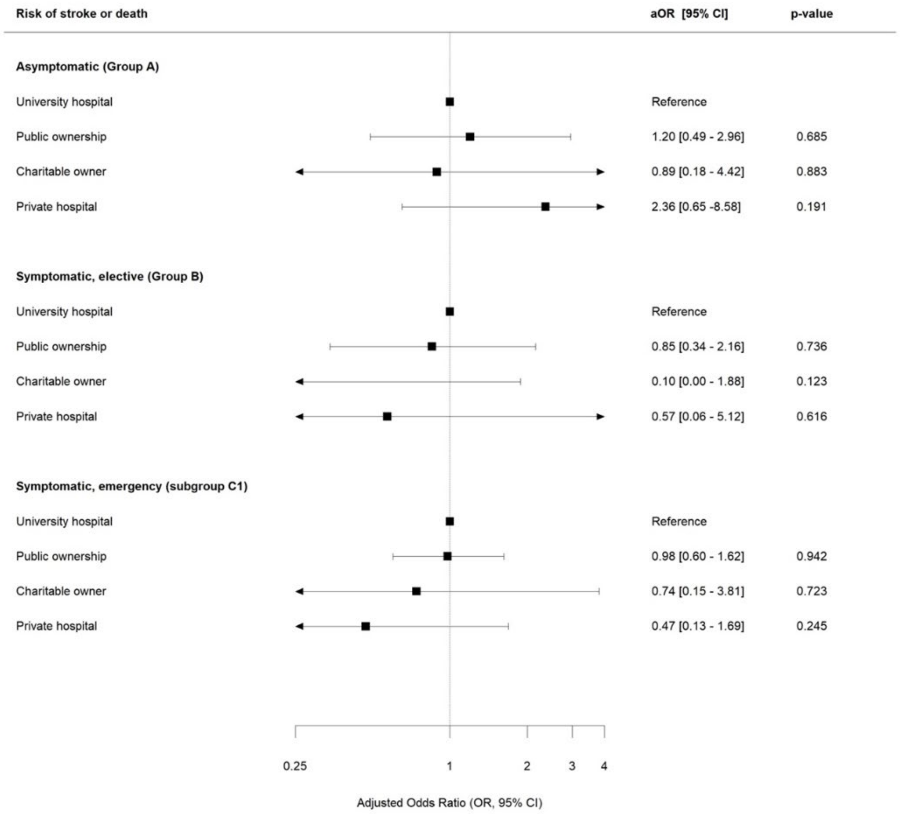

aOR = odds ratio adjusted for age, sex, ASA, ipsi- and contralateral degree of stenosis, pre- and post-procedural assessment by neurologist, and annual caseload of treating centre. In indication group B, additional adjustment for neurological symptoms and time interval between index event and treatment.

Supplemental Text:

Limitations:

This is a secondary data analysis and thus, all shortcomings of observational studies using routine data must be considered in principle. These limitations were discussed in detail elsewhere and will be summarized here:

- First, the study design was retrospective and observational. Because patients were not randomized to the hospitals, selection bias as well as confounding by indication is possible. Furthermore, active or passive selection of patients by the hospital as well as selection of hospitals by the patients or the doctors advising them (e. g. general practitioner) could lead to selection bias. Although this selection (possible cherry-picking) is precisely the core of the analysis, this implies that all results need to be interpreted as associations rather than causal relationships. Nevertheless, the sometimes significant differences identified between the individual hospital groups are precisely the interesting results of this study and not a bias.
- Second, follow-up data covered only the in-hospital period. Because most of the perioperative events presumably occur within the first days after CEA or CAS, a detection bias is considered to be low. However, it must be remembered that it is not the short-term results that are important for the quality of medical care, but the long-term results, e. g. the sustainable prevention of strokes, which unfortunately cannot be answered with this legal database.
- Third, all data in the database are self-reported by the attending physicians, and reporting bias cannot be ruled out. However, data reports were reviewed by the regional offices for quality assurance (Landesgeschäftsstellen für Qualitätssicherung) and the occurrence of suspect data induced a process of structured dialogue to clarify abnormalities systematically. Nonetheless, underreporting of adverse events is theoretically probable and might be the reason for the overall low rates of perioperative stroke or death reported in this registry. Although underreporting cannot be ruled out, any potential information bias can be considered homogeneous among the variables analysed in this study.
- Fourth, ASA stage was the only variable besides age, sex, degree of stenosis, symptom status and others that could be used for risk adjustment. Unfortunately, other important characteristics were simply not available in the database by law. Thus, although the ASA stage was only used for a very rough and of course far from adequate risk adjustment, it was the best that was possible with the available database. Furthermore, residual confounding cannot be excluded, because some possible confounders were not available (e.g., information on the type of embolization protection device or stent used, comorbidities, cardiovascular risk profile, routine medication, presence of restenosis, intraoperative

heparin or protamine application, or the reasons for the application of a certain procedural technique). Additionally, unobserved confounders as well as selection bias caused by patient choice or regional differences (e. g. driving distances to the next hospital) may have influenced the results [38].

- Fifth, for data protection reasons, the unique hospital identifier is pseudonymised when the data are transmitted to the IQTIG, so that linking the level 1 data with those of levels 2 and 3 was unfortunately only possible for Bavaria.
- Last, unfortunately, the revenue for individual patients is not documented in the clinical quality assurance data. The specific remuneration for patients is calculated based on the main hospital diagnosis, secondary diagnoses, procedures and other factors. Unfortunately, this important economic factor was not available for the analysis.
